# *Batrachochytrium dendrobatidis* Shows High Genetic Diversity and Ecological Niche Specificity among Haplotypes in the Maya Mountains of Belize

**DOI:** 10.1371/journal.pone.0032113

**Published:** 2012-02-28

**Authors:** Kristine Kaiser, John Pollinger

**Affiliations:** Department of Ecology and Evolutionary Biology, University of California Los Angeles, Los Angeles, California, United States of America; University of California Riverside, United States of America

## Abstract

The amphibian pathogen *Batrachochytrium dendrobatidis* (*Bd*) has been implicated in amphibian declines around the globe. Although it has been found in most countries in Central America, its presence has never been assessed in Belize. We set out to determine the range, prevalence, and diversity of *Bd* using quantitative PCR (qPCR) and sequencing of a portion of the 5.8 s and ITS1-2 regions. Swabs were collected from 524 amphibians of at least 26 species in the protected areas of the Maya Mountains of Belize. We sequenced a subset of 72 samples that had tested positive for *Bd* by qPCR at least once; 30 samples were verified as *Bd*. Eight unique *Bd* haplotypes were identified in the Maya Mountains, five of which were previously undescribed. We identified unique ecological niches for the two most broadly distributed haplotypes. Combined with data showing differing virulence shown in different strains in other studies, the 5.8 s - ITS1-2 region diversity found in this study suggests that there may be substantial differences among populations or haplotypes. Future work should focus on whether specific haplotypes for other genomic regions and possibly pathogenicity can be associated with haplotypes at this locus, as well as the integration of molecular tools with other ecological tools to elucidate the ecology and pathogenicity of *Bd*.

## Introduction

The amphibian pathogen *Batrachochytrium dendrobatidis* (*Bd*), a chytrid fungus, is globally distributed [1] and has been implicated in the declines of over 200 amphibian species [2]–[4]. Although previous work has suggested that the genetic diversity of *Bd* is low across a variety of coding and noncoding genomic regions [5]–[6], consistent with the hypothesis that it is an emerging pathogen, various authors have also reported differences in virulence [7]–[8] or phenotype [9] among strains. For example, *Bd* infects some species and populations without causing mortality [10], [11] while causing dramatic declines in others [2]. In addition, some authors [6], [12] have found substantial diversity in the ITS and 5.8 s genomic regions, the loci used for detection of *Bd* by qPCR, but no exploration of the significance of such diversity has been undertaken.

It is clear that in order to understand chytridiomycosis, the disease caused by *Bd*, more information on the diversity and ecology of *Bd* is needed. As a result, several authors have used models to gain further insights into the bioclimatic factors which may limit or predict the distribution of *Bd* [13]–[16]. Unfortunately, these authors have lacked genetic diversity data which would allow them to evaluate separate haplotypes of *Bd*. Thus, all observations of *Bd*-positive samples have, by necessity, been treated as equals with regard to habitat niche. However, haplotypes with different virulence and biology [7], [8] may have dissimilar ecology.

Thus, our study had two goals. The first was to characterize the range and diversity of *Bd* in the Maya Mountains of Belize. *Bd* is almost ubiquitous in Central Americ [2], [17]–[18], and Cheng et al. [19] suggest that the fungus first entered the region in the 1980's. However, the amphibians of Belize have never been tested for *Bd*, and little is known about their population status. We used qPCR detection of the 5.8 s – ITS1-2 region to detect *Bd*, and sequenced positive samples. We used the genotypic diversity found in the 5.8 s and ITS regions to examine phylogenetic relationships and divergence in *Bd* in Belize. The second goal was to determine whether different haplotypes of *Bd* are distributed similarly in the environment, and if the same bioclimatic and remote sensing predictors would equally predict their distributions. We found that the pathogen was ubiquitous in the Maya Mountains, with divergent haplotypes across the range. We used habitat niche modeling using the two most common *Bd* haplotypes [20] to predict potential distributions and identify which bioclimatic factors were influential for their distributions. Niche-modeling results indicated that the different haplotypes occupied disparate habitat niches.

## Results

We tested a total of 524 swabs from at least 25 species of frogs and salamanders in triplicate for the presence of *Bd* by qPCR (Table S1). Of these, 86 (16%) tested positive at least once in the three tests. All field sites tested positive for *Bd*, suggesting that the pathogen is broadly distributed in this region. Of the subset of qPCR products that had tested positive for *Bd* at least once and that we sequenced (n = 72), 31 sample sequences (including a positive control, isolate JEL423 collected from Panama) were positive for Bd.

**Figure 1:**
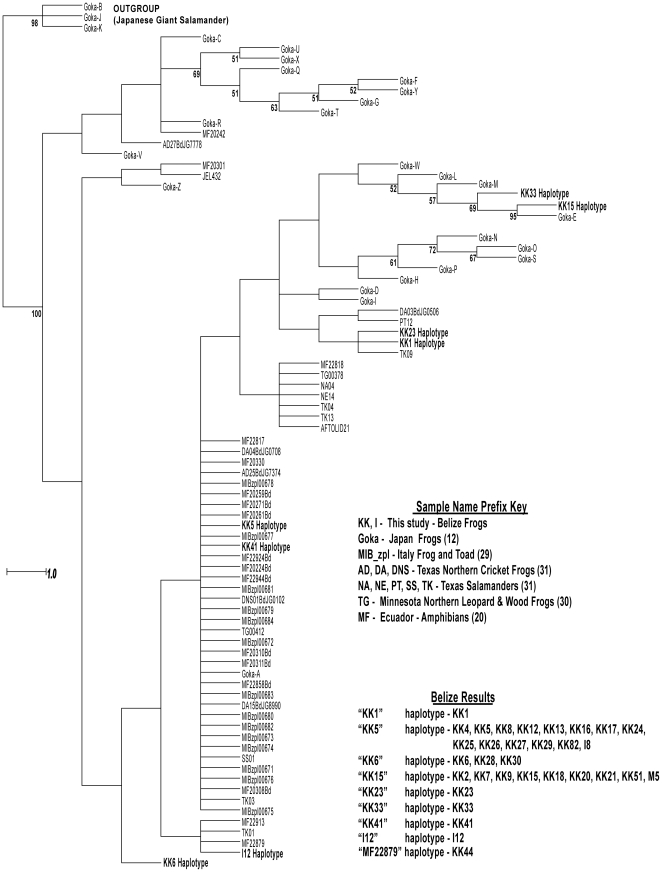
Phylogenetic tree for ITS haplotypes of *Bd* using maximum parsimony analysis. Parsimony bootstrap support values where values are greater than 50% are shown under branches. Haplotypes identified in other recent surveys of frogs in Japan (12); frogs in Italy (29); frogs and salamanders in Texas (32), unspecified amphibians in Ecuador (19) and frogs in Minnesota (30) are shown along with haplotypes identified in this study on frogs from Belize (highlighted in yellow). The tree is rooted with *B. dendrobatidis* outgroup haplotypes observed in Japanese giant salamanders. Positive control isolate JEL423 is haplotypes KK5/Goka A.

We found a total of 8 unique *Bd* haplotypes (Table S1). Two of these have been previously identified by [12] as haplotypes A (KK5 in this study) and E (KK15 in this study), one by [21] as MF22879 (I12 in this study;19); all others are unique, previously undescribed haplotypes (KK1, KK6, KK23, KK33, and KK41; Genbank accession numbers xxx–xxx)(Fig. 1). KK5 and KK15 were the most commonly represented haplotypes (Table S1). KK5 was represented by fourteen samples from at least seven different species of amphibian from five taxonomic families and was detected at twelve sites. Haplotype KK15 was represented by nine samples from at least five species from four families and detected at eight sites. KK6 was found three times; all other haplotypes were found only once.

**Figure 2:**
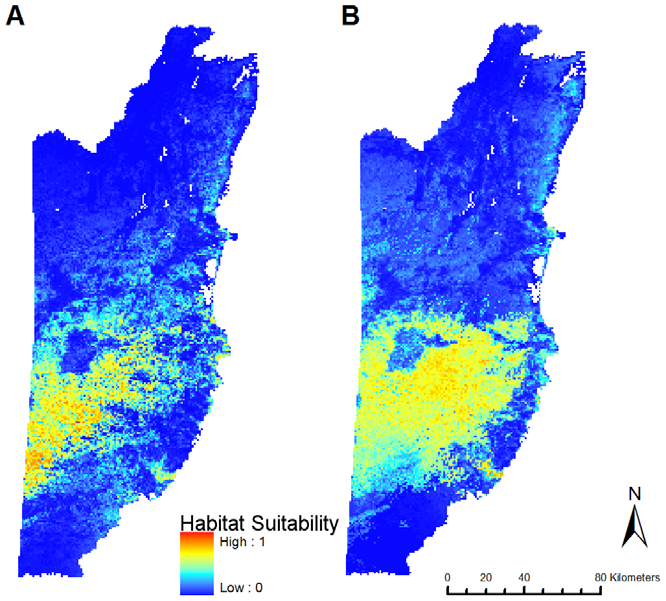
Maxent distribution models for *Bd* haplotypes. Predicted distributions shown are from cross-validated models. A. Haplotype KK5. B. Haplotype KK15.

Predicted distributions for the two *Bd* haplotypes overlapped substantially, with the predicted distribution of KK5 being more limited than that of KK15 (Fig. 2). Jackknife results [22] for both haplotypes showed models were successful in predicting distributions and were statistically significant with both MPT and T10 thresholds (KK5: *P* = 0; KK15, *P* = 0.000008). Models differed dramatically among haplotypes in the specific factors that had the highest relative contribution to the model (Table 1). Removal of vegetation parameters from the models had no effect on the qualitative results of models (Table 2): KK5 distribution was explained primarily by temperature variables. Although KK15 was driven primarily by precipitation, there was a slight increase in temperature contribution to models.

**Table 1:** Relative variable contribution to distribution models.

Variable	KK5 Contribution (%)	KK15 Contribution (%)
Tree Cover	31.69	33.55
Annual Mean Temperature	23.35	3.41
Mean Diurnal Temperature Range	6.45	15.15
Precipitation Warmest Quarter	0.56	23.03

Only variables with a relative contribution of 15% to at least one haplotype are included.

**Table 2:** Relative variable contribution to distribution models with vegetation parameters removed.

Variable	KK5 Contribution (%)	KK15 Contribution (%)
Annual Mean Temperature (BIO1)	52.82	8.61
Warmest Month Maximum Temperature (BIO5)	18.20	21.02
Warmest Quarter Precipitation (BIO18)	1.10	34.36
Mean Diurnal Temperature Range (BIO2)	12.81	22.17

Relative contribution of variables to models with no vegetation predictors. See online supporting materials for explanation of variables. Only variables with a relative contribution of 15% to at least one haplotype are included. Although the variables which contribute most to each model change slightly with the inclusion of vegetation, haplotype KK5 remains determined primarily by temperature factors, while KK15 is driven most by precipitation. See text for details.

Maxent gives heuristic estimates of relative contributions of each variable to models. Although annual temperature (Bioclim variable BIO1, Table S2) was the biggest contributor to the KK5 distribution model (52.82%, Table 1), the variable jackknife procedure demonstrated that Mean Diurnal Temperature Range (BIO2) yielded the most unique information not present in other variables. BIO2 contributed only 12.8% to the model, but was the third highest contributor to the KK5 model. In all but one KK5 model, Maximum Temperature of the Warmest Month (BIO5) had the highest gain when used as an isolated variable, despite not contributing the most to the model. BIO5 thus can be considered the single most informative variable. In the single case where BIO5 did not have the highest gain, BIO1 was both the largest contributor to the model and the variable with the highest gain.

KK15 models also found that BIO5 was generally the most informative variable; in all but two cases, this was true. In the two models where BIO5 did not have the highest gain, Warmest Quarter Precipitation (BIO18) and Precipitation Seasonality (BIO15) did. BIO18 was the variable with the most unique information.

Subsample models were reasonably predictive [23]: the KK5 distribution model average test AUC was 0.871±0.074 (standard deviation). The KK15 model average test AUC was 0.891±0.068. Subsampled models for both haplotypes converged on the same predictors as cross-validated models.

## Discussion

Sequenced samples of the broadly-distributed pathogen *Bd* contained eight unique 5.8 s – ITS1-2 haplotypes of *Bd*, five of which were previously undescribed sequences. Moreover, habitat-niche modeling found that two *Bd* haplotypes had significantly different associations with ecological variables. The observed association of both haplotypes with vegetation cover may simply be an artifact as virtually all of our samples came from sites within parks with relatively high broadleaf forest cover; removal of vegetation measures from the model had no qualitative effect on the model, confirming that the haplotype distributions were not entirely dependent on vegetation cover. The slight increase in reliance on temperature for haplotype KK15 after removal of vegetation parameters may simply reflect a correlation between temperature and vegetation. Although the habitats surveyed in this study would not be considered primary or pristine forest [24]–[25], no sites were undergoing active deforestation and were generally intact; thus *Bd* prevalence would not likely be depressed by habitat loss as in other regions in Central America [26]. Areas with lower forest cover in the Maya Mountains (e.g., the Mountain Pine Ridge Reserve, which experienced a substantial decrease in forest cover due to beetle infestation) exist and are subject to different climatic regimes. Whether *Bd* occurs in these regions is currently not known, but if so, further study is warranted to determine whether haplotype distributions follow similar patterns there.

Heuristic estimates of variable contributions showed that annual temperature contributed the most to the model; however, jackknife of variables demonstrated that finer-scale variables such as maximum temperature of the warmest month and mean diurnal temperature range were more informative for both KK5 and KK15 distribution models. These results concur with those of Rohr and Raffel's climatic variability hypothesis [27]. Precipitation data for this region are unfortunately available only at quarterly intervals; future research in regions where fine-scale climatic data should would benefit from haplotype distribution modeling with such data.

Although maximum temperature of the warmest month had the highest gain in almost all models, this may represent a constraint at the level of the organism, rather than a character specific to haplotype distribution: temperatures in this region prior to the onset of the rainy season can approach 40C. Although *Bd* is tolerant of a wide range of temperatures, its thermal optimum is between 17–25C, and at temperatures above 30C, survivorship appears to decrease substantially [28]. Aside from the importance of warmest month maximum temperature, the divergence between variable contributions in KK5 and KK15 models is a novel and potentially important finding: if different haplotypes assort based on different environmental parameters, this will have implications for ecological and distributional modeling, as well as epidemiology.

Although molecular data has been used previously in modeling [29]–[30], this is the first use of such data to model the ecological distributions of different haplotypes. Genotypic differences in *Bd* among populations using nuclear SNP markers have been previously correlated to differences in protein expression and virulence [8]. Although it has been suggested [6] that the ITS region of *Bd* is highly variable within and among individuals, and thus is not suitable for population comparisons, the fact that such pronounced ecological differences in predicted distributions have been found suggests that the observed diversity in the 5.8 s - ITS region locus may be indicative of diversity at conservative nuclear loci that may be diagnostic at the population level. This potential diversity should be investigated for correlation to the spectrum of *Bd* virulence to which Belizean amphibians are currently exposed. Moreover, combined with multiple reports of differing virulence among strains [7]–[8] or lineages [31], our results suggest that there may be substantial differences among populations or haplotypes that we do not yet fully understand, including ecological niche partitioning. We thus recommend that sequencing of other *Bd* genomic regions associated with virulence [8] should be performed and compared with corresponding 5.8 s-ITS haplotypes for the same samples, to examine whether pathogenicity can be associated with specific 5.8 s-ITS haplotypes. In summary, the integration of molecular tools with techniques such as habitat niche distributional modeling using haplotypes is critical to elucidating the ecology of *Bd* and ultimately providing insight into its pathogenicity.

## Materials and Methods

This study was carried out in strict accordance with the recommendations in the Guide for the Care and Use of Laboratory Animals of the National Institutes of Health. All research was conducted under UCLA IACUC permit (2008–009–01B) and under permit from the Belize Forest Department (permits CD/60/3/08(15), CD/60/3/08(36), CD/60/3/08(02)) and the Belize Agricultural Health Authority (Certificate No. 34736).

### Field Sampling

We sampled amphibians at a variety of sites from the protected areas of the Maya Mountains from June–August, 2006–2008. Skin swabs were taken from pelvic patches, legs, and toes using sterile cotton swabs (Dynarex) and stored in 70% ethanol. Clean gloves were used for each frog. Frogs were released at point of capture.

### Extraction of DNA from Swabs

Control and sample swab tips were twice agitated with 0.5 mm dia. silica beads (Biospec, Bartlesville, OK) in PBS using a Qiagen tissue disruptor (Tissue Lyser II) for 45 s at 30 Hz, and centrifuged at 8 krpm for 1 minute. The liquid was then removed and subjected to a standard extraction protocol for cell wall biochemical lysis and DNA extraction using a Qiagen QIAamp DNA Minikit.

### qPCR assay and sequencing

The *Bd* qPCR assay was performed in triplicate using the ITS-1 Chytr and 5.8 S Chytr primers of [32] and high resolution melt detection with Roche High Resolution Melting Master reagents (Roche Diagnostics, Indianapolis, IN). Results were analyzed using ABI 7900 HT Sequence Detection Systems for Windows XP (version 2.3) and High Resolution Melting (version 2.0) software. qPCR HRM product from positive samples were run on 1% agarose gels. Target size products were excised from the gels, cleaned using a standard ZymoClean (ZymoResearch Corp, Irvine, CA) PCR product cleanup protocol, and used in an ABI BigDye 3.1dye terminator sequencing product preparation reaction. Sequencing reactions were carried out with both ITS1 Chytr and 5.8 S Chytr primers. Products were sequenced on an ABI3730XL sequencer (Applied Biosystems, Carlsbad, CA).

### Sequence Alignment and Phylogenetic Analysis

Sequence chromatograms were manually reviewed and base calls verified using the software program Bioedit (version 7.0.5.3; 28). All 5.8 S/ITS *Bd* sequences currently in NCBI Genbank [74; see 12 for list] were added to the sequences generated in this study and the hypervariable noncoding ITS regions containing significant insertions/deletions manually aligned. Unique haplotypes were identified by comparison with the previously published *Bd* ITS region sequences. We selected the *Bd* ITS region sequences for the Japanese giant salamander (*Andrias japonicas*) per [12] as an outgroup for the phylogenetic analyses. Phylogenetic analyses were conducted on the set of Belize *Bd* haplotypes verified as *B. dendrobatidis* and the set of 74 amphibian haplotype sequences available from Genbank [12], [21], [33]–[35]. PAUP (version 4.0b) was used to reconstruct the phylogenetic trees. Indels were coded as binary data, and all characters were weighted equally. We performed a maximum parsimony analysis using heuristic search with the TBR branch-swapping option and a bootstrap analysis with 1,000 replications.

### Ecological Niche Distribution Modeling

We used Maxent v. 3.3.1 [20], a maximum entropy-based species distribution prediction modeling tool, to create haplotype distribution models. Sixteen climatic and remote-sensing data variables were included in the models (Table S2). All layers were at 1 km^2^ resolution. Recommended default values for the model and features were used, including the removal of duplicate records. No test cases were used within Maxent. Two different thresholds were used for the purposes of assessments: the Minimum Presence Threshold (MPT) and T10 [22]. All data were used to train the model, and we incorporated the jackknife method and associated p-value computational tool (version 2/20/06) to obtain a non-biased measure of the performance of the model [22]. In addition, Maxent's Subsample option was used. One hundred models were generated, using 20% of data each run as test data. All other options were left as default.

## Supporting Information

Table S1 **Species sampled and** ***Bd*** **prevalence.**
(DOC)

Table S2 **Climatic and environmental layers included in models.**
(DOC)
