# Correction: *Batrachochytrium dendrobatidis* Shows High Genetic Diversity and Ecological Niche Specificity among Haplotypes in the Maya Mountains of Belize

**DOI:** 10.1371/annotation/fe06ff76-bdd0-41d9-be11-07b9646d0ca8

**Published:** 2012-08-09

**Authors:** Kristine Kaiser, John Pollinger

GenBank policy as of 2011 is to not accept sequences <200 bp long (The GenBank Submissions Handbook. National Center for Biotechnology Information, Bethesda, MD; 2011). Thus the author has provided sequences of the newly identified haplotypes in a new Supplemental Table S3. Table S3 can be viewed here: 
