# Supplementary material for: Batrachochytrium dendrobatidis Shows High Genetic Diversity and Ecological Niche Specificity among Haplotypes in the Maya Mountains of Belize
Source: PLoS One. 2012 Feb 28;7(2):e32113. doi: 10.1371/journal.pone.0032113 (PMC3289636; doi:10.1371/journal.pone.0032113)
Supplement: Table S1 — Species sampled and Bd prevalence. (DOC) [file pone.0032113.s001.doc]

Table S1. Species sampled and *Bd* prevalence.

| **Species** | **Number of samples** | **Haplotypes (occurrences)** |
| --- | --- | --- |
| **Order Caudata** |  |  |
| **Family Plethodontidae** |  |  |
| *Bolitoglossa mexicanum* | 1 |  |
| *Bolitoglossa rufescens* | 17 |  |
|  |  |  |
| **Order Anura** |  |  |
| **Family Bufonidae** |  |  |
| *Incilius campbelli* | 13 | KK15(1) |
| *Incilius marinus* | 3 |  |
| *Incilius sp* | 7 | KK5(1); MF22879(1) |
| *Incilius valliceps* | 20 |  |
| **Family Centrolenidae** |  |  |
| *Hyalinobatrachium fleischmanni* | 4 | KK5(1) |
| **Family Hylidae** |  |  |
| *Agalychnis callidryas* | 22 |  |
| *Agalychnis moreletii* | 55 | KK5(1); KK6(1); KK33(1) |
| *Dendropsophus ebraccatus* | 63 | KK15(1) |
| *Dendropsophus microcephalus* | 39 |  |
| *Smilisca baudinii* | 16 |  |
| *Smilisca cyanosticta* | 6 | KK5(2) |
| *Smilisca sp.* | 1 |  |
| *Tlalocohyla loquax* | 13 |  |
| *Tlalocohyla picta* | 13 |  |
| **Family Craugastoridae** |  |  |
| *Craugastor chac* | 79 | KK41(1); I12(1) |
| *Craugastor laticeps* | 9 |  |
| *Craugastor loki* | 4 |  |
| *Craugastor sabrinus* | 26 | KK5(1); KK15(2) |
| *Craugastor sandersoni* | 18 |  |
| *Craugastor sp.* | 5 |  |
| **Family Eleutherodactylidae** |  |  |
| *Eleutherodactylus leprus* | 1 |  |
| **Family Microhylidae** |  |  |
| *Gastrophryne elegans* | 1 |  |
| **Family Ranidae** |  |  |
| *Lithobates berlandieri* | 4 | KK5(1) |
| *Lithobates juliani* | 21 | KK6(1); KK15(2) |
| *Lithobates vaillanti* | 26 | KK1(1); KK5(4); KK15(2); KK6(1) |
| **Family Rhinophrynidae** |  |  |
| *Rhinophrynus dorsalis* | 4 |  |
|  |  |  |
| *Species data unavailable* | 33 | KK5(1); KK15(1); KK23(1) |
| *Control* (JEL423) | 1 | KK5(1) |
| **Total** | 524 |  |

Number of samples refers to total number of swabs for a given species. The final column lists which haplotypes were found on each species and how many of each were found. Species-level identification was unavailable for 33 swabs provided by others. Haplotype KK5 is synonymous with (12) haplotype A; KK15 with haplotype E. MF22879 sequence refers to (19).
