# Supplementary material for: Batrachochytrium dendrobatidis Shows High Genetic Diversity and Ecological Niche Specificity among Haplotypes in the Maya Mountains of Belize
Source: PLoS One. 2012 Feb 28;7(2):e32113. doi: 10.1371/journal.pone.0032113 (PMC3289636; doi:10.1371/journal.pone.0032113)
Supplement: Table S2 — Climatic and environmental layers included in models. (DOC) [file pone.0032113.s002.doc]

Table S2. Climatic and environmental layers included in models.

| **Environmental Layer** | **Layer Description** |
| --- | --- |
| BIO1 | Annual Mean Temperature (*10) |
| BIO2 | Mean Diurnal Range (Mean of monthly (max temp - min temp)) |
| BIO4 | Temperature Seasonality (standard deviation *100) |
| BIO5 | Max Temperature of Warmest Month |
| BIO6 | Min Temperature of Coldest Month |
| BIO12 | Annual Precipitation |
| BIO15 | Precipitation Seasonality (Coefficient of Variation) |
| BIO16 | Precipitation of Wettest Quarter |
| BIO17 | Precipitation of Driest Quarter |
| BIO18 | Precipitation of Warmest Quarter |
| BIO19 | Precipitation of Coldest Quarter |
| Tree Cover | Percentage of Tree Cover (2001) |
| NDVI Max | Normalized Difference Vegetation Index; A measure of spatial pattern of vegetation density (2001) |
| NDVI Range Mean | Normalized Difference Vegetation Index; Mean of wettest half of year minus mean of driest half of year: A measure of vegetation seasonality (2001) |
| EVI Max | Enhanced Vegetation Index; A measure of spatial pattern of vegetation density (2001) |
| EVI Range Mean | Enhanced Vegetation Index; Mean of wettest half of year minus mean of driest half of year: A measure of vegetation seasonality (2001) |

Remote sensing layers are derived from MODIS and available from the Global Land Cover Facility at the University of Maryland. For discussion of the differences between the remotely sensed layers, see Saatchi (31). All layers were at a resolution of 1 km^2^.
