# Supplementary figures and images for: Correction: Batrachochytrium dendrobatidis Shows High Genetic Diversity and Ecological Niche Specificity among Haplotypes in the Maya Mountains of Belize
Source: PLoS One. 2012 Aug 9;7(8):10.1371/annotation/fe06ff76-bdd0-41d9-be11-07b9646d0ca8. doi: 10.1371/annotation/fe06ff76-bdd0-41d9-be11-07b9646d0ca8 (PMC3435121; doi:10.1371/annotation/fe06ff76-bdd0-41d9-be11-07b9646d0ca8)

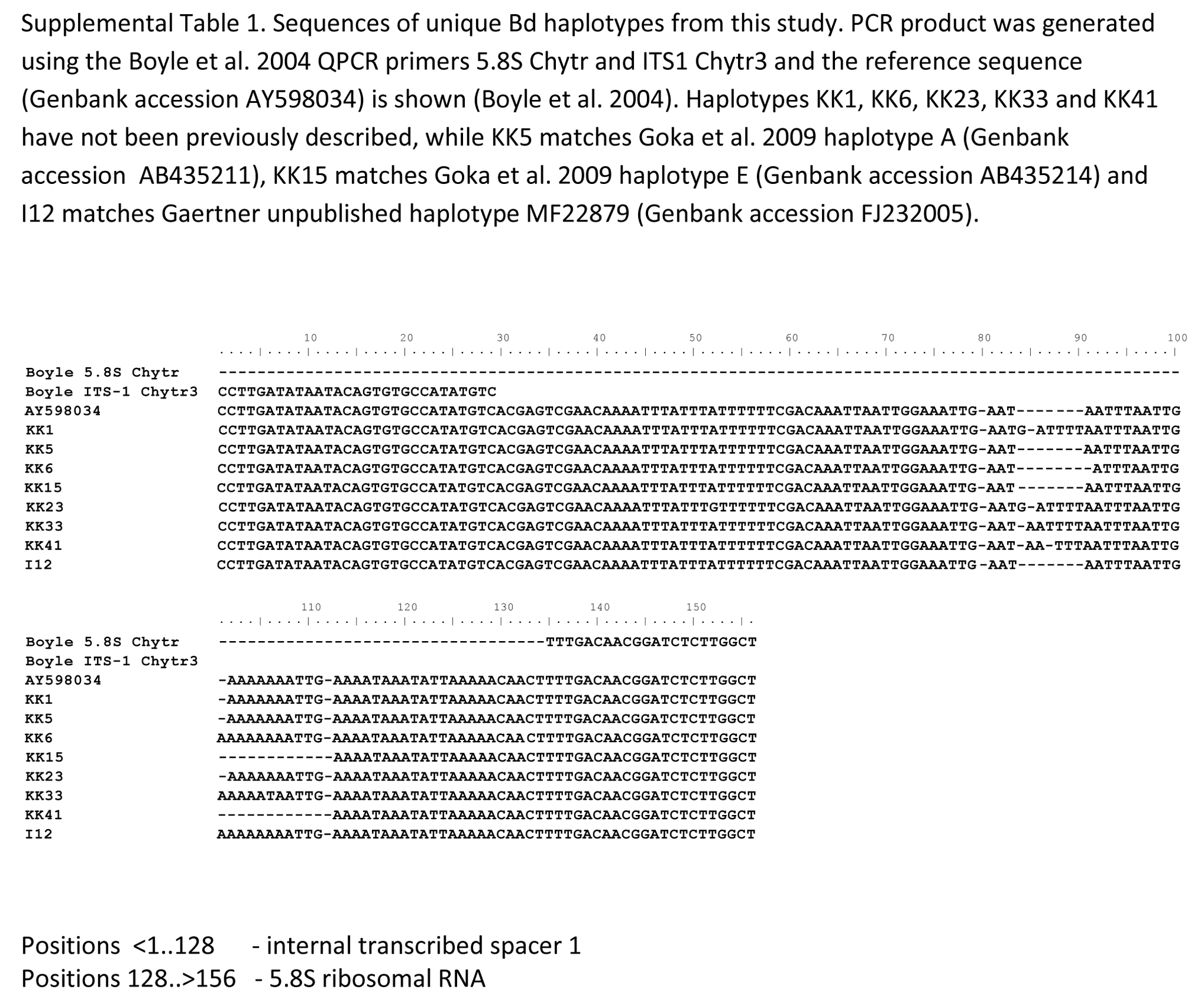

Supplement: Supplementary file 1 [file pone.fe06ff76-bdd0-41d9-be11-07b9646d0ca8.s001.tif]
